# Modeling the public health impact of malaria vaccines for developers and policymakers

**DOI:** 10.1186/1471-2334-13-295

**Published:** 2013-07-01

**Authors:** Julia K Nunes, Vicky Cárdenas, Christian Loucq, Nicolas Maire, Thomas Smith, Craig Shaffer, Kårstein Måseide, Alan Brooks

**Affiliations:** 1 PATH Malaria Vaccine Initiative, 455 Massachusetts Avenue NW, Suite 1000, Washington, DC 20001, USA; 2 Current address: Aeras; 1405 Research Boulevard, Rockville, MD 20850, USA; 3 Epidemiology and Public Health Department, Swiss Tropical and Public Health Institute, Socinstrasse 57, 4051, Basel, Switzerland; 4 University of Basel, Petersplatz 1, CH-4003, Basel, Switzerland; 5 Applied Strategies, 951 Mariners Island Blvd., Suite 400, San Mateo, CA 94404, USA; 6 London School of Hygiene and Tropical Medicine, Keppel Street, London WC1E 7HT, UK; 7 Current address: Norad, Norwegian Agency for Development Cooperation, Ruseløkkveien 26, P.O. Box 8034 Dep, NO 0030, Oslo, Norway; 8 PATH Malaria Vaccine Initiative; Bâtiment Avant Centre, 13 chemin du Levant, 01210, Ferney-Voltaire, France; 9 Current address: 5023 DLF City Phase 4, Gurgaon 122009, Haryana, India; 10 Current address: International Vaccine Institute, SNU Research Park, San 4-8; Nakseongdae-dong; Gwanak-gu, Seoul 151-919, Korea

## Abstract

**Background:**

Efforts to develop malaria vaccines show promise. Mathematical model-based estimates of the potential demand, public health impact, and cost and financing requirements can be used to inform investment and adoption decisions by vaccine developers and policymakers on the use of malaria vaccines as complements to existing interventions. However, the complexity of such models may make their outputs inaccessible to non-modeling specialists. This paper describes a Malaria Vaccine Model (MVM) developed to address the specific needs of developers and policymakers, who need to access sophisticated modeling results and to test various scenarios in a user-friendly interface. The model’s functionality is demonstrated through a hypothetical vaccine.

**Methods:**

The MVM has three modules: supply and demand forecast; public health impact; and implementation cost and financing requirements. These modules include pre-entered reference data and also allow for user-defined inputs. The model includes an integrated sensitivity analysis function. Model functionality was demonstrated by estimating the public health impact of a hypothetical pre-erythrocytic malaria vaccine with 85% efficacy against uncomplicated disease and a vaccine efficacy decay rate of four years, based on internationally-established targets. Demand for this hypothetical vaccine was estimated based on historical vaccine implementation rates for routine infant immunization in 40 African countries over a 10-year period. Assumed purchase price was $5 per dose and injection equipment and delivery costs were $0.40 per dose.

**Results:**

The model projects the number of doses needed, uncomplicated and severe cases averted, deaths and disability-adjusted life years (DALYs) averted, and cost to avert each. In the demonstration scenario, based on a projected demand of 532 million doses, the MVM estimated that 150 million uncomplicated cases of malaria and 1.1 million deaths would be averted over 10 years. This is equivalent to 943 uncomplicated cases and 7 deaths averted per 1,000 vaccinees. In discounted 2011 US dollars, this represents $11 per uncomplicated case averted and $1,482 per death averted. If vaccine efficacy were reduced to 75%, the estimated uncomplicated cases and deaths averted over 10 years would decrease by 14% and 19%, respectively.

**Conclusions:**

The MVM can provide valuable information to assist decision-making by vaccine developers and policymakers, information which will be refined and strengthened as field studies progress allowing further validation of modeling assumptions.

## Background

Modeling can provide key input into public health decisions to use, or not use, new health technologies in the developing world [1,2]. Models provide data on a given intervention’s impact, cost-effectiveness, and/or financing requirement estimates. Models allow analyses of situations that are difficult or impossible to replicate in real life, including in field trials, such as the absolute impact of a new malaria control intervention in the absence of any existing interventions. They can provide insight by analyzing complex scenarios and identifying which are most likely to occur and which parameters, and their ranges, are the most influential [3]. It is important that modeling estimates be made available to support evidence-based decision-making. This paper describes a new model for vaccines against malaria, a disease that caused approximately 660,000 deaths in 2010, mostly of children in sub-Saharan Africa [4]. The Malaria Vaccine Model (MVM) was designed to assist vaccine developers and policymakers in developing countries and in global organizations to make informed decisions about the design and adoption of malaria vaccines.

The demand for evidence on which policymakers and developers can base decisions is increasing. Those developing new, often more expensive, public health interventions for use in developing countries must invest in interventions with the appropriate attributes (e.g., level of efficacy, costs, mode of delivery) to realize desired health impacts. Such decisions will need to be supported by modeled estimates, such as potential impact and financial requirements. The GAVI Alliance (GAVI) has invested close to $100 million since 2000 in activities related to *Haemophilus influenzae* type B (Hib), pneumococcal conjugate, and rotavirus vaccines. Establishing the value of these vaccines through the generation of impact estimates was one of the key activities, which arose from the recognition that multi-year delays occurred in the introduction of Hib vaccine by countries, in part because of the lack of data on the burden of disease and potential impact of vaccines [5]. Ensuring that global, regional, and country decision makers have access to these data in advance could improve the timeliness with which future interventions reach those in need.

A number of models have recently been used to estimate the impact of interventions worldwide. Some were intended to inform global policies and have focused on individual vaccines, such as human papilloma virus, HIV, and rotavirus vaccines [6-8]. By contrast, the Lives Saved Tool (LiST) estimated the impact of up to dozens of child survival interventions (including malaria control interventions) across 42 low- and middle-income countries worldwide [9]. This model, which was intended to help global policymakers prioritize interventions, has been extended for use in individual countries. The ProVac Initiative in the Americas included a model to support country decision-making on the use of new vaccines; the London School of Hygiene and Tropical Medicine and the Swiss Tropical and Public Health Institute (Swiss TPH) developed a web-based tool built on modeled data to assist African policymakers in making local decisions on the use of intermittent preventive treatment of malaria in infants [10,11]. These examples illustrate the importance of being clear about a model’s purpose and target audiences, and of taking into account the impact of multiple interventions against diseases.

A malaria vaccine model needs to inform vaccine developers as well as policy and financing decision-makers at global, regional, and country levels. Among other requirements, it needs to be linked to data on transmission and epidemiology in each country and allow for the consideration of malaria vaccines in the context of other malaria interventions available to countries. Malaria vaccine models were recently reviewed by the World Health Organization (WHO) [12]. There are two published dynamic models that estimate the potential impact of malaria vaccines. One model has been under development since 2003 at the Swiss TPH [13]. The second, more recent, model was developed at Imperial College London [14]. Both models consider the dynamics of malaria transmission and of natural immunity to *Plasmodium falciparum* using simulation approaches to reflect the underlying relationship between interventions and how much disease they may be able to avert. These approaches can be difficult for non-modeling specialists and policymakers to utilize. The WHO encourages policymakers to include model based cost-effectiveness estimates in support of eventual malaria vaccine adoption decisions [15]. However, published economic analyses of malaria vaccination based on such models [16] so far have not considered supply-side considerations, such as manufacturing capacity, which influence implementation and ultimately impact on health.

With global efforts to develop malaria vaccines showing promise, the PATH Malaria Vaccine Initiative (MVI) worked with partners to develop a Malaria Vaccine Model (MVM) that extended the Swiss TPH model by including supply-side considerations and country-specific estimates, and that allowed vaccine developers and policymakers to more easily access the outputs of the Swiss TPH model. The MVM was designed to fill a range of roles for different audiences. For non-profit organizations working on vaccine development, the MVM can help explore the trade-offs in potential product characteristics as part of informing and prioritizing R&D decisions. The MVM can inform global and regional policymakers of the potential impact associated with various delivery strategies and the financing needs for malaria vaccines. At the country level, it can assist local policymakers, for example, through targeted, facilitated workshops, in understanding the potential impact of malaria vaccines in the context of existing malaria control interventions.

The first version of the MVM was developed with the Boston Consulting Group and Swiss TPH and utilized by MVI and partners between 2005 and 2007 to inform discussions regarding the establishment of an Advance Market Commitment for malaria vaccines [17]. The current version of the MVM, presented here, was developed between 2008 and 2010 by MVI in collaboration with Swiss TPH and Applied Strategies. This version reflects the understanding gained from experience with the initial version about underlying model parameters and design (including outputs), potential uses, and software. The MVM uniquely integrates supply, demand, and cost with public health impact estimates generated from a dynamic malaria vaccine model, while presenting both country-specific and global level data in a user-friendly interface.

This paper describes the major design features, critical parameters, and outputs of the MVM. A demonstration scenario was created that is used to illustrate the model functionality and is not intended to estimate the impact of any specific malaria vaccine. While the modeled vaccine efficacy is substantially higher than the most clinically advanced malaria vaccine candidate today [18], this demonstration scenario models a hypothetical vaccine based on a strategic goal for the development of a malaria vaccine set by the international community in 2006 through the Malaria Vaccine Technology Roadmap: the development of a vaccine with protective efficacy of more than 80% against clinical disease and lasting longer than four years [19]. The results of this demonstration scenario are presented, along with lessons learned for the future development of similar models.

## Methods

### Overview of model structure

**Figure 1:**
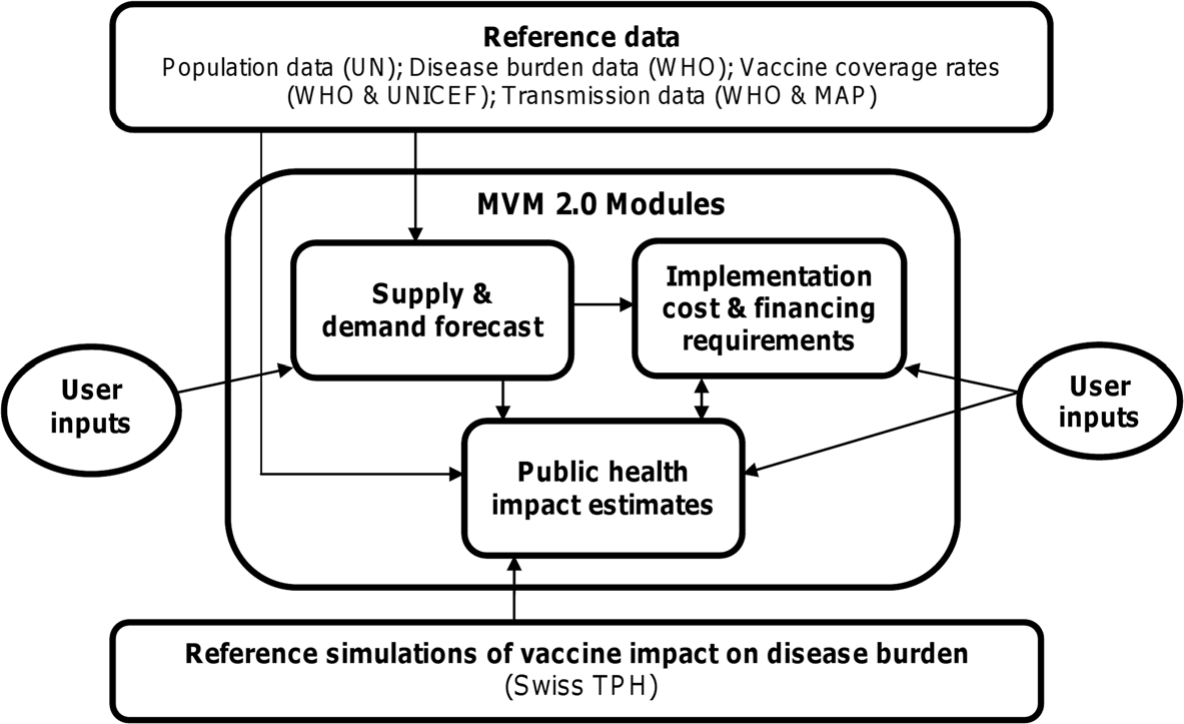
**Structure of the Malaria Vaccine Model (MVM).** This figure depicts the MVM structure. The central box contains the three modules within the MVM: supply and demand forecast, public health impact estimates, and financial analysis. Above and below the MVM modules box are two rectangles describing the underlying reference data accessed by the modules. Arrows represent the interaction between reference data and user inputs and modules within the MVM, as well as between modules of the MVM.

The MVM is composed of three distinct modules (Figure 1). The first module, modeling vaccine supply and demand, was developed by MVI and Applied Strategies. The second module, based on Swiss TPH’s model simulations, estimates the public health impact expected from different malaria vaccines deployed in varying populations through several modes of delivery. The third module uses data from the first two modules and adds vaccine price and cost of delivery to estimate the total investment that would be required to achieve the potential public health gains and costs per event averted. Each module includes a built-in sensitivity analysis function for some key parameters (e.g. vaccine price, year of vaccine availability).

As described above, one demonstration scenario using a high efficacy vaccine was selected to illustrate the model’s functionality. Descriptions of each module are provided below, followed by descriptions of the input values used in the demonstration scenario.

### Software and interface

MVM was built as a Microsoft® Windows desktop application using the Microsoft®. NET framework (version 3.5). The criteria considered in the software selection process, drawing on experience from the first version of the model, were ease of use by non-experts, familiarity (similar to Microsoft Windows), ability to interface well with large datasets containing elements from the Swiss TPH model, and ease of updating new reference datasets. Microsoft Excel was used for the first version of the model, however it was challenging to efficiently manage the complex data underpinning the model, and it was less user-friendly. Although the Microsoft®. NET software is proprietary, an unrestricted license was granted for use in the MVM.

Model inputs are either selected from drop-down menus or entered as numbers. The outputs include graphics displayed on the screen, with an option to export data into Excel for customized reports.

### Supply and demand forecast

Based on user inputs and reference data, the supply and demand forecast module predicts the quantity of vaccines that will be available at a given time and the number of doses that are in demand by countries (Tables 1 and 2).

**Table 1:** Supply and demand module: user inputs for supply parameters

**Parameter name**	**Definition**	**Input values**
Manufacturing capacity	Describes current known or estimated facility capacity.	Number of doses
	Describes timeline of new facilities.	Year available
Year of vaccine approval	The user determines the year of approval by a national regulatory authority (NRA) and World Health Organization (WHO) prequalification, which are assurances of vaccine quality as a prerequisite for availability. The user then selects whether NRA approval or WHO prequalification is required by each country.	Year

**Table 2:** Supply and demand module: user inputs for demand parameters

**Parameter name**	**Definition**	**Input values**
Year of vaccine approval	The user determines the year of approval by a national regulatory authority (NRA) and World Health Organization (WHO) prequalification, which are assurances of vaccine quality as a prerequisite for availability. The user then selects whether NRA approval or WHO pre-qualification is required by each country.	Year
Years between vaccine approval and country adoption	Identifies time between the first year that a vaccine is available and the year that each country implements a vaccine.	Number of years
Maximum coverage	Describes the largest percentage of the target population reached in each country. The user can either define the percentage reached, or use the default settings referencing the actual coverage levels of other vaccines.	%
Years for each country to reach maximum coverage	Describes the number of years between implementation and achievement of maximum coverage.	Number of years
Number of doses per regimen	Describes the number of doses required to fully vaccinate each person at efficacy levels described under the public health impact module.	3 or 4
Vaccine wastage	Describes the proportion of doses that will not be administered. Vaccine wastage is a percentage set by the user, but suggestions are provided in the MVM based on the number of doses per vial, according to WHO projected vaccine wastage (http://www.who.int/immunization_delivery/systems_policy/logistics_projected_wastage/en/index.html; accessed: 2011 Apr 28). The model does not take buffer stock (a one-time 25% increase of vaccine doses distributed in a logistics system in the first year of implementation) into account.	%
Target population	The age group in which the vaccine is used: infants (represented by the annual birth cohort for each country), 5-17 month olds, 1 year olds (yos), 0-4 yos, 1-4 yos, 1-39 yos, 5-39 yos, or the total population	Age range
Product preference	In the case of multiple available vaccines, the user may model scenarios in which particular countries prefer one vaccine over another.	Product name
Maximum acceptable price	The maximum price a country is willing to pay for a vaccine.	USD

All countries with an annual average birth cohort estimate and projection from the Population Division of the Department of Economic and Social Affairs of the United Nations (UN) as of 2008 were included in the MVM. Results can be generated for both individual and multiple countries at once. Users may create classifications and/or groupings of these countries for a particular analysis. For example, users could select only those countries that are GAVI-funding eligible, they may focus on high malaria disease burden countries in sub-Saharan Africa, or they may generate estimates for a single country.

#### *Reference data*

**Population** Data on the population of each country (birth cohort, one-year olds, 0-4 year olds, 0-39 year olds, and the total population) were drawn from the UN Population Division [20], and are regularly updated with the latest data, thereby accounting for changes in demographic trends. Users may select the age range of the target population consistent with the expected vaccination strategies of potential malaria vaccines (Table 2).

**Vaccine coverage** The MVM included historical and projected vaccine coverage for Bacille Calmette-Guerin; first, second, and third doses of diphtheria-tetanus-pertussis (DTP1, DTP2, DTP3); and measles-containing vaccine for each country. Users may set the maximum vaccine coverage for each country equal to historical data for any of the above vaccines, or they may set maximum vaccine coverage at any level from 0–100%. Data on the 2005–2007 coverage rates were obtained from the WHO and United Nations Children’s Fund (UNICEF) [21]. Future coverage levels for this analysis were based on projections from WHO (Lara Wolfson, WHO ICE-T v4.0, Oct 2007, unpublished data).

#### *Module summary and demonstration scenario*

Supply estimates are based on user inputs describing each manufacturer’s anticipated capacity, timing of expected increases in capacity, and year of vaccine availability, assuming development success and regulatory approval. Demand estimates in the form of total doses required per year are based on the size of the target population in each country, the maximum level of coverage and time taken to reach this level, and the number of doses in a regimen. Along with the above inputs and reference data, the model outputs include the year the vaccine is available for use in each country (from both supply and approval perspectives), the number of doses demanded, and the number of doses available to meet demand in any given year.

For the demonstration scenario, it was assumed that supply would not be constrained by manufacturing capacity and that all countries would require vaccine prequalification by WHO. Forty countries in sub-Saharan Africa that experienced a malaria disease burden in 2006 of 100 deaths per year or greater, or that experienced a malaria mortality rate of 10 deaths per 100,000 per year or greater, were included. The criteria were intended to allow inclusion of large countries with many cases at a relatively low rate, and small countries with few absolute cases but which have a significant rate relative to the population size. The time horizon modeled in the demonstration scenario was 10 years of vaccine use, beginning in 2016 (see Additional file 1).

**Figure 2:**
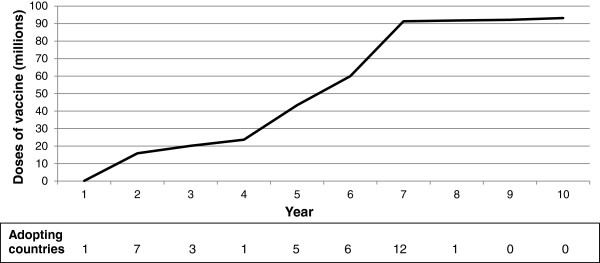
**Estimated number of malaria vaccine doses delivered per year over a 10-year period.** Numbers of doses delivered are based upon reports of the use of Hib vaccine to WHO between 2001 and 2010 from 40 countries in sub-Saharan African with significant malaria burden. The number of countries that began implementation each year is indicated below the x axis (note that there is a delay between the time each country begins implementation and reaches peak coverage).

Demand in the demonstration scenario (Figure 2) assumed a vaccine regimen of three doses delivered to a target population of infants and a wastage rate of 10% (see Additional file 1). No product preference or maximum acceptable price was modeled. Years between vaccine approval and country adoption of the modeled malaria vaccine, maximum coverage, and the time to reach maximum coverage were benchmarked from historical data, based on the uptake of Hib vaccine from 2001 to 2010 (either on its own, in the form of a tetravalent vaccine with DTP, or as a pentavalent vaccine with DTP and hepatitis B). WHO data on Hib coverage served as a reference point for the number of years post vaccine availability that each country began implementation, each country’s maximum coverage (DTP3 coverage was selected to represent maximum), and the time it took for Hib3 to match DTP3 levels [22]. Since malaria is more commonly recognized as a health threat than Hib, and because countries have gained experience in vaccine implementation since Hib introduction began, unadjusted data from Hib introduction might therefore underestimate the uptake of a malaria vaccine. The data were adjusted such that all countries that did not adopt Hib in the first two years of its availability were modeled as adopting a malaria vaccine two years earlier than they adopted Hib. The time each country took to reach maximum coverage was not adjusted. If countries did not introduce Hib by 2010, they were excluded from the demonstration scenario. The exception was Nigeria, which appears to be moving more quickly to adopt more recent vaccines than it did with Hib [23]. Nigeria was assumed to adopt the malaria vaccine midway through the time horizon analyzed, and reach maximum coverage in two years (see Additional file 2).

### Public health impact estimates

Based on user inputs and reference data on the transmission level of malaria in each country and the selected vaccine characteristics, the principal outputs of the public health impact module are the estimates of uncomplicated cases, severe cases, deaths, and DALYs that could be averted by vaccine use.

The natural history, epidemiology of *P. falciparum* and vaccine impact were modeled using a stochastic simulation model developed at Swiss TPH and described in detail in previous publications [24-29]. The age structure of the simulated human populations was based on the number of people in each of a set of standard age groups in the demographic surveillance site of Ifakara, Tanzania [30]. Swiss TPH model parameters were estimated by fitting the model to field data from a variety of settings across sub-Saharan Africa [31]. Pre-vaccination transmission intensity was scaled to give the different required values of the initial exposures (e.g. 0.1, 1, 10, or 100 infected bites per person per annum as described below). Pathogenesis and case management (including hospitalization of severe cases) were also simulated as described in previous publications [26,29,32]. Any changes in transmission intensity induced by a vaccination program (due to herd immunity or other indirect effects) were captured, but the vectorial capacity (a mosquito population’s capacity to transmit malaria) followed the identical periodic pattern as in the absence of vaccine. The current version of the MVM utilized the key parameters found to be of interest to developers and policymakers in the first version of the MVM - for example, specific populations in specific transmission settings and utilization of particular modes of delivery (Table 3).

**Table 3:** Public health module user inputs

**Parameter name**	**Definition**	**Input values**
**Type of vaccine**	Describes the antigens targeted. Options include pre-erythrocytic (PE), blood-stage (BS), or a combination of these plus a vaccine component targeting sexual, sporogonic, and/or mosquito (SSM) antigens to interrupt transmission from an infected person to the next.	Select one: PE, BS, PE + BS, PE + SSM, BS + SSM, PE + BS + SSM
**Vaccine efficacy**	Describes the efficacy of the vaccine immediately after completing the full regimen. For a pre-erythrocytic vaccine, efficacy against infection is defined as the proportional reduction in incidence of blood-stage infection. The user selects the level of efficacy against clinical disease, which is lower than efficacy against infection (and the model implements a mapping between the two). For a blood-stage vaccine, efficacy is defined as the proportional reduction in blood-stage parasite density. For a vaccine targeting the SSM antigens, efficacy is defined as the proportion by which the probability that a mosquito is infected during one bite is reduced [33].	Select one: 35%, 50%, 60%, 75%, 85%
**Decay rate of efficacy against infection**	Describes the time after vaccination at which the vaccine protection against infection is half of its initial value. Decay rate of efficacy assumes an exponential decay of efficacy.	Select one: 2, 4, 10 years
**Future malaria transmission**	Transmission is described as the percent of the population of the country of interest residing in each of five categories of entomological inoculation rate (EIR), which is a measure of how many infectious bites a person receives per year (ibpa) in a given setting. The starting transmission level for each country is derived from the reference data described below. The user can choose to keep transmission fixed at this level throughout the time period under consideration, or can enter scenarios of future transmission, for example, specific to an individual country.	See Additional file 3
**Mode of vaccine delivery**	Describes the means by which the vaccine is delivered to its target population. The options include routine vaccination via a country’s routine immunization system and campaign delivery.	See Table 4
**Booster compliance rate**	Percentage of population originally vaccinated who receive a single booster dose.	%

#### *Reference data*

**Disease burden** Malaria-specific morbidity and mortality rates were obtained from the WHO for both the entire population and the population under 5 years of age [34] and are updated annually. In situations where no data specific to the under-5 population were available, the total population’s malaria morbidity and mortality rates were applied. Projections of future disease burden were based on each country’s current morbidity rate or current mortality rate multiplied by the country’s population forecast for the selected population. Options for changing the future disease burden, such as from implementation of other interventions, are described below. Estimates of vaccine impact on disease burden are based on the levels of transmission in each country, as described below.

**Malaria transmission** In the MVM, the intensity of transmission is represented by the entomological inoculation rate (EIR), defined as the product of the human biting rate of mosquitoes and the proportion of mosquitoes that are infectious. EIR is measured in the number of infective bites per person per annum (ibpa). Two sources of data on transmission were incorporated into the MVM, allowing the user to choose between them. Data from WHO were in the form of the percentage of each country’s population at high or low risk for malaria infection [34]. Alternatively, the Malaria Atlas Project (MAP) data were in the form of *P. falciparum* parasite prevalence [35]. The WHO source data were not provided in the form of EIR, which is the required input for transmission for the Swiss TPH model. The data were converted to EIR using methodology endorsed by both MAP and an external expert panel (see Additional file 3). Fortunately, much progress has been made recently in understanding the relationship between different measures of transmission [36]. The MVM calculates the percentage of each country’s population that falls into each of five EIR levels (0, 0.1, 1, 10, and 100 ibpa). Since transmission can vary multifold between settings and the means of measurement are imprecise [37], the EIR levels listed above were selected to clearly reflect this wide variation. The user is also free to input future changes in levels of transmission for countries, for example based upon the expected impact of other malaria interventions.

#### *Vaccine impact on disease burden*

Estimates of the impact of vaccines on disease burden were based on the parameters of and simulations run in the previously published, dynamic model of the Swiss TPH, and integrated with country-specific transmission data in the MVM. Pre-erythrocytic vaccination was simulated as described in previous publications [33,38], assuming that vaccination leads to both a reduction in the proportion of inoculations from the bites of infected mosquitoes and the resulting blood-stage infections. This leads to values of efficacy against infection higher than the proportion of clinical episodes prevented by such a vaccine [39]. Blood-stage vaccination was simulated by assuming that the vaccine reduced blood-stage parasite densities while transmission-blocking vaccines reduce the proportion of blood feeds that result in infection of the vector [33]. Duration of protection against infection is modeled as an exponential decay in vaccine efficacy as previously described [40], and the decay rate of efficacy against infection is the key variable that was chosen as a user input in the MVM. Decay rate of efficacy against infection describes the time after vaccination at which the vaccine protection against infection is half of its initial value. Vaccination scenarios were paired with non-vaccination comparator simulations to provide impact estimates of the outcomes found to be of greatest interest from the first version of the MVM: the numbers of cases (uncomplicated and severe), deaths, and disability-adjusted life years (DALYs) averted through vaccine use over time periods of interest.

MVI, Applied Strategies, and Swiss TPH agreed on possible combinations of input parameters anticipated to be of interest to vaccine developers and policymakers, resulting in over 100,000 scenarios to be simulated (Table 4). Each scenario was run multiple times, and mean frequencies of events were computed in order to reduce stochastic variability in the estimates of health effects. The results of these simulations are stored in look-up tables as reference data in the MVM, drawn upon as necessary according to the particular combinations of inputs for specific populations in the scenario chosen by the user (see Additional files 4, 5, 6, 7, 8, 9, 10, 11, 12, 13, 14, 15 and 16).

**Table 4:** Vaccination strategies generated by Swiss TPH

**Strategy name**
No vaccination
Routine infants with a boost 2 years later
Routine infants (no boost)
Routine infants with a boost 2 years later PLUS a catch-up of 5–17 month olds (no boost)
Routine infants (no boost) PLUS a catch-up of 5–17 month olds (no boost)
Routine infants with a boost 2 years later PLUS a catch-up of 1–5 year olds (no boost)
Routine infants (no boost) PLUS a catch-up of 1–5 year olds (no boost)
Routine infants with a boost 2 years later PLUS a catch-up of 1–39 year olds (no boost)
Routine infants (no boost) PLUS a catch-up of 1–39 year olds (no boost)
Routine 5–17 month olds with a boost 2 years later
Routine 5–17 month olds (no boost)
Periodic 1–5 year olds every 5 years (no boost) PLUS a catch-up of 6–39 year olds (no boost)

**Legend**: Strategies reflect different implementation approaches and target population. “Infants” mean children approximately six weeks of age at first vaccination. “Routine” vaccination means that it is happening continuously as individuals reach the target age. “Catch-up” campaign means a one-time, mass vaccination targeting the indicated age range. “Periodic” campaign means regular mass vaccinations targeting the indicated age range, at the indicated frequency.

#### *Module summary and demonstration scenario*

Estimates of the public health impact of a vaccine are generated based on the type of vaccine, initial efficacy and decay rate of vaccine efficacy against infection, mode of vaccine delivery, and transmission setting. The model outputs from this module are the total number of events averted (uncomplicated cases, severe cases, deaths, and DALYs) in the total population or those less than 5 years of age through use of the vaccine.

For the demonstration scenario, although none of the current vaccine candidates in trials is expected to meet this target, a pre-erythrocytic vaccine with an efficacy of 85% against uncomplicated malaria cases was assumed in light of the strategic goal for malaria vaccine development set in 2006 [19]. The decay rate of efficacy against infection was four years. These values of efficacy and its decay were selected for the demonstration scenario as they were expected to be broadly consistent with targets set by the international community [19]. Routine infant immunization (via the Expanded Program on Immunization) was selected as the mode of delivery. As described above, the user enters the projected transmission in each country in the form of EIR into the MVM. For the demonstration scenario, three different projections were created to reflect decreases in transmission from current levels, potentially due to implementation of existing interventions. The projection applied to the demonstration scenario used the MAP-derived EIR distribution across countries, shifting one-quarter of the population at each EIR level (0, 0.1, 1, 10, and 100 ibpa) into the next lower category (e.g., from EIR of 10 down to 1). The other two projections shifted either one-half or three-quarters of the population at each level to the next lower level of EIR. It was assumed that the percentage of the population residing in each EIR level decreased in a linear fashion to the next lowest level over a period of five years, and remained constant for the remaining five years modeled.

### Implementation cost and financing requirements

The implementation cost and financing requirements module estimates the total investment that would be required to purchase and deliver the vaccine simulated in the public health impact module, and the cost of each event averted in 2011 US dollars. User inputs are presented in Table 5.

**Table 5:** Financial module user inputs

**Parameter name**	**Definition**	**Input values**
**Vaccine price**	The cost of the vaccine in US dollars (USD) as set by the manufacturer, including insurance and delivery to the airport of a country specified by the consignee.	$
**Cost of injection equipment and disposal**	The cost of the syringes and waste disposal equipment.	$
**Cost of vaccine delivery**	Different costs can be entered for each country, allowing customized estimates that take into account factors such as the cold-chain capacity and transport needs of each.	$
**Discount rate**	A distinct discount rate can be entered for the supplier and the donor, reflecting the cost of capital of each.	%
**Financing scenario**	Users select the financing start and end dates.	Years
	Users select the level of country co-pay to complement support from donor organizations. Users can create various financing scenarios.	USD

#### *Reference data*

This module does not include pre-entered reference data.

#### *Module summary and demonstration scenario*

Based on the user inputs of vaccine price and implementation costs, the module generates the total investment required to achieve the public health gains estimated in the public health impact module, and calculates the cost per case, severe case, death, and DALY averted. Depending on the assumed financing scenario, for example, if donors support a portion of the purchase and implementation costs, the model provides a break-down by donor and country contributions for each year of vaccine use. A discount rate can be used to calculate the present values of the total investments, and can be summed to estimate the net present value.

The demonstration scenario assumed a vaccine price remaining constant at $5/dose, including insurance and delivery to the airport of a country as specified by the consignee. Inflation was not included. This price is broadly consistent with the assumed cost of other new vaccines for low-income countries, such as pneumococcal conjugate and human papilloma virus vaccines (as there is no price yet determined for any potential malaria vaccine). In addition to the vaccine price, the implementation cost modeled was $0.33/dose and injection equipment costs were $0.07/dose, totaling $1.20/fully immunized child. Costs were based upon a simulation study of the cost of introducing a malaria vaccine into the routine immunization system in Tanzania [41], and were also consistent with results from a similar study following the introduction of a pentavalent DTP-hepatitis B-Hib vaccine in Ethiopia [42]. Results from the demonstration scenario are presented without discounting and with a discounting of up to 5%, to cover a range around the most commonly used rate of 3% in the literature [43].

Table 6 summarizes the assumed values and vaccine characteristics for the demonstration scenario that were described throughout the methods section. Assumptions related to cold chain and personnel were not separately costed for the demonstration scenario, but instead based upon published analyses which built upon experience with existing vaccines (e.g. DTP-HepB-Hib) [42,44].

**Table 6:** **Summary of values** &**vaccine characteristics in the demonstration scenario**

**Parameter name**	**Assumed value**	**Rationale or source, as applicable**
**Supply and demand module**		
Time period modeled	10 years	Mid-range period that is long enough to allow for countries to implement and observe impact; a longer period would increase model uncertainty
Year of vaccine approval	2016	Date in the medium term selected to avoid the increased model uncertainty associated with longer time horizons
Countries	40 African countries with high disease burden	100 deaths/year or malaria mortality rate of 10 deaths per 100,000 per year or greater [45]
Years between vaccine approval and country adoption	0-10, based on each country’s adoption of *Haemophilus influenza* type B (Hib) vaccine (see Methods)	See Methods under “Supply and demand forecast”
Maximum coverage	3rd dose of diphtheria-tetanus-pertussis vaccine (DTP3) level of each country, as projected (see Methods)	Demonstration scenario assumes routine vaccination (below); DTP3 coverage is therefore a realistic estimate of what might be achieved
Years for each country to reach maximum coverage	1 to 3, based upon each country’s adoption of Hib vaccine (see Methods)	See Methods under “Supply and demand forecast”
Number of doses per regimen	3	Consistent with other, licensed vaccines
Vaccine wastage	10% (assumed 2 doses/vial)	Consistent with other, licensed vaccines
Target population	Infants	Infants carry the greatest burden of disease [20]
**Public health module**		
Type of vaccine	Pre-erythrocytic	The most advanced candidate is a pre-erythrocytic vaccine and therefore the most likely type to first reach 85% efficacy
Vaccine efficacy against clinical disease	85%	Consistent with the strategic goal of the 2006 Malaria Vaccine Technology Roadmap
Decay rate of efficacy against infection	4 years	Consistent with the strategic goal of the 2006 Malaria Vaccine Technology Roadmap
Future malaria transmission	¼ of the population in each Entomological Inoculation Rate category shifted to the next lowest by 2020 (0, 0.1, 1, 10, 100)	Assumes continued scale-up of other interventions and progress toward global targets
Mode of vaccine delivery	Routine infant immunization (Expanded Program on Immunization (EPI))	Infants carry the greatest burden of disease and are routinely vaccinated via the EPI system
Booster compliance rate	Not used in demo. scenario	None assumed
**Financial module**		
Vaccine price	$5/dose	There is no price yet determined for any potential malaria vaccine; consistent with the cost of other new vaccines for low-income countries
Cost of injection equipment and disposal	$0.07/dose	See Methods section under Implementation cost and financing requirements [42,44,46]
Cost of vaccine delivery, including:	$0.33/dose	Consistent with experience with pentavalent vaccine in Ethiopia [42,44,46]
• Cold chain requirement of 2-8°C		
• Personnel and training		
Discount rates	0% and 5%	Consistent with the full range of rates used in the sub-Saharan context [43]

### Sensitivity analysis

The MVM allows the user to perform sensitivity analyses to determine the magnitude of the impact of select factors on the model outputs. Sensitivity-analysis outputs can be generated for any given year, or over the entire period modeled. Sensitivity-analysis results indicate the impact of the adjusted parameter(s) on the public health and financial estimates. The following model inputs can be increased or decreased by a percent of the value set by the user: country willingness to pay (maximum price); discount rate; price of vaccine or injection equipment; and percentage of product wastage. Country adoption and product availability inputs can be increased or decreased by a number of years relative to a baseline. Alternatively, for those parameters not included in the formal sensitivity analysis, such as vaccine efficacy, decay rate of efficacy, transmission, or the use of a booster dose, model outputs from several scenarios can be saved and compared.

## Results

The demonstration scenario was run to illustrate the functionality of the MVM. The inputs selected for this scenario were described above in the Methods section. The outputs of the demonstration scenario are presented below (see Additional file 17).

### Supply and demand module

Based on the historical data from Hib, adapted as described above, in its first two years of use, over 16 million (M) doses of malaria vaccine were required to meet demand, plateauing at 93 M between years 7 and 10 (Figure 2). Over the course of 10 years of vaccine use, the MVM outputs from the demonstration scenario indicated that a total of 532 M doses would be required in the 40 African countries in the analysis.

### Public health impact module

The estimated number of doses of vaccine required from the supply and demand module was used as an input for the calculation of public health impact. While the underlying calculations were made for each country, the results were presented at an aggregate level to give a sense of impact across all of sub-Saharan Africa.

**Figure 3:**
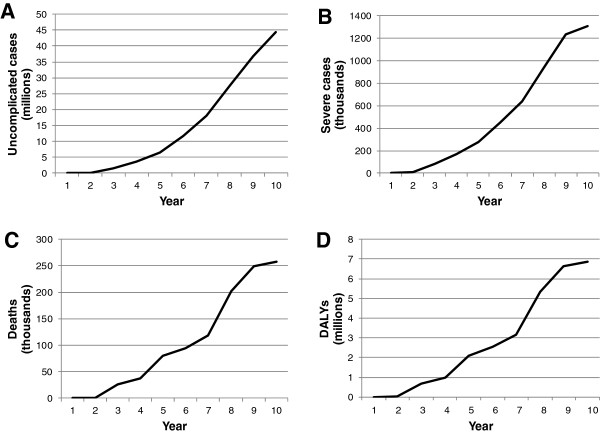
**Annual malaria events averted in 40 high-burden African countries by the simulated vaccine.** MVM projections of the number of **A)** uncomplicated cases; **B)** severe cases; **C)** deaths; **D)** DALYs averted by use of an 85% efficacious pre-erythrocytic vaccine with an efficacy decay rate of 4 years over 10 years in sub-Saharan Africa.

Based on the demonstration scenario inputs, an estimated 150.4 M uncomplicated cases of malaria would be averted over 10 years of vaccine use (Table 7), with 44.3 M averted in year 10 alone (Figure 3). Over this same period, 5.1 M severe cases of malaria would be averted, along with 1.1 M deaths and 28.4 M DALYs. The number of severe cases, deaths, and DALYs averted annually increased over the whole time period, with the largest number seen in the final year: 1.3 M, 258,000, and 6.9 M, respectively (Figure 3). It can be useful to present these data as ratios of the number of events averted per 1,000 vaccine recipients, as well. Averaged over the 10-year time period, the inputs for the demonstration scenario led to 943 uncomplicated cases averted/1,000 vaccinees, 32 severe cases averted/1,000 vaccinees, 7 deaths averted/1,000 vaccinees, and 178 DALYs averted/1,000 vaccinees (Table 7).

**Table 7:** Cumulative number and ratio of malaria events averted, and cost per event averted

	**Total events averted over 10 years (M)**	**Events averted/1,000 vaccinees**	**Cost/event averted (undiscounted, 2011 USD)**	**Cost/event averted (discounted, 2011 USD)**
**Uncomplicated malaria**	150	943	19	11
**Severe malaria**	5	32	561	309
**Death**	1	7	2,690	1,482
**DALY**	28	178	101	56

### Implementation cost and financing module

The cost per event averted was calculated in the MVM for each year of the analysis using the non-discounted total investment (vaccine price plus injection equipment plus delivery costs). It could also be informative to consider the present value of the investments, calculating the cost per event averted with a discounted number. These data are presented in Table 7 as the aggregate over the entire period. In the demonstration scenario, the undiscounted cost was, in 2011 US dollars, $19 per uncomplicated malaria case averted, $561 for each severe case averted, $2,690 for each death averted, and $101 for each DALY averted. After discounting the total investments at 5% to a net present value in 2011 US dollars, the costs were $11, $309, $1,482, and $56, respectively.

### Sensitivity analysis

**Figure 4:**
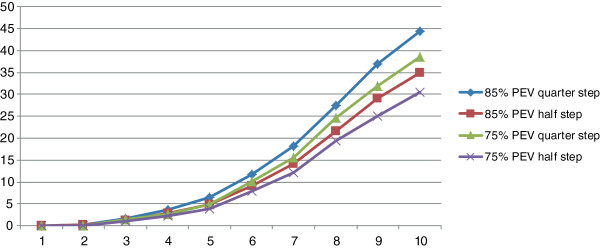
**Implications of changing vaccine efficacy or transmission intensity on impact.** The impact of vaccine efficacy and transmission setting on the potential number of uncomplicated malaria cases averted annually over a 10-year period of vaccine use. The demonstration scenario of 85% efficacy and a transmission setting in which the risk level (EIR category) of one-quarter of the population at risk was reduced averted the most cases (●), followed by 85% efficacy at a setting in which the risk level of one-half the population at risk was reduced (**▲**), 75% efficacy at the transmission setting in which one-quarter of the population experienced reduced risk (■), and 75% efficacy at a transmission setting in which one-half the population at risk experienced a reduction in transmission (×).

Lower vaccine efficacy and a larger decrease in malaria transmission were considered (Figure 4 and Table 8). Reducing the efficacy to 75% decreased the estimated number of uncomplicated cases averted over 10 years by 14%, from 150 M to 130 M. Similarly, the total number of severe cases, deaths, and DALYs averted were predicted to decrease by 14%, 19%, and 18%, respectively (data not shown). While the total investment would remain the same, fewer events would be prevented, increasing the undiscounted (and discounted) cost/event averted from $19 ($11) per uncomplicated case to $22 ($12). The cost per severe case averted would increase to $652 ($359), per death averted to $3,340 ($1,840), and per DALY averted to $124 ($68).

**Table 8:** Impact of efficacy and transmission setting on the number and cost of events averted

	**% decrease in public health impact (events averted)**	**% increase in cost/event averted**	**Increase in cost/case averted 2011 USD, undiscounted (discounted)**
**Uncomplicated cases**			
Efficacy	13	16	3 (2)
Transmission	21	26	5 (3)
Both	33	47	9 (5)
**Severe cases**			
Efficacy	14	16	91 (50)
Transmission	21	26	145 (80)
Both	31	46	258 (142)
**Deaths**			
Efficacy	19	24	650 (358)
Transmission	21	26	711 (392)
Both	36	57	1528 (842)
**DALYs**			
Efficacy	18	22	23 (12)
Transmission	21	26	27 (14)
Both	35	54	55 (30)

**Legend:** Table shows the results from the scenarios modeled as part of the sensitivity analysis. Percentage decrease or increase in public health impact or cost is relative to the demonstration scenario results, presented in Table 7. “Efficacy” refers to a decrease in the vaccine efficacy from 85% in the demonstration scenario to 75%. “Transmission” refers to a change in future transmission from the demonstration scenario, in which one-quarter of the population in each category of risk was shifted to the next lowest category, to the sensitivity analysis, in which one-half the population was at reduced risk of infection. “Both” refers to a scenario in which both efficacy and transmission setting were decreased as described above.

Great progress has been made over the past decade in scaling up the current malaria control interventions [4]. While our demonstration scenario shifted one-quarter of the population in each EIR category to the next lower level of risk, it is possible that national malaria control programs and their partners will achieve greater success in lowering transmission. To understand the potential public health gains that could be attributed to the vaccine in a setting with lower transmission, and therefore many fewer cases to avert, we also modeled the scenario described in the Methods section in which one-half of the population in each EIR category is shifted to the next lowest level of risk. MVM outputs indicated that such a shift would lead to approximately 21% fewer events averted across all categories over the 10-year period, relative to the demonstration scenario.

## Discussion

This paper describes a collaborative effort to develop a malaria vaccine model of supply, demand, public health impact, and costs designed for use by diverse audiences at country, regional, and global levels and based upon simulations of vaccine impact on public health outcomes from the Swiss TPH model. The functionality of the model was demonstrated by estimating the impact and implementation costs associated with a hypothetical vaccine broadly consistent with international targets. Modeled estimates suggest that such a malaria vaccine could have an important public health impact, and it will be important to compare the model functionality and results with those from other malaria vaccine models as they become available.

The model was designed to have the flexibility to include user inputs, which could allow for the incorporation of new data as they become available. For example, the user can input any scenario for future malaria transmission, whether an increase or decrease, which takes advantage of the ever-improving ability of models to estimate the impact of other interventions on transmission. Another example of user-entered data illustrated in the demonstration scenario was the use of historical data from the implementation of the Hib vaccine as inputs for country adoption.

Future versions of the model could be applied to other types of malaria vaccines that are being considered or that are currently in research and development—for example, future versions of the model could include different efficacy levels, delivery strategies, or additional target populations, such as pregnant women. The most clinically advanced vaccine candidate is RTS,S, in development by GlaxoSmithKline (GSK) and MVI. Results of the RTS,S Phase III trial for efficacy and safety, indicate that 3 doses of the RTS,S vaccine candidate reduced clinical and severe malaria over the first year of follow-up by 56% and 47%, respectively, in children 5-17 months of age at first vaccination; and 31% and 37%, respectively, in infants 6-12 weeks of age at first vaccination [18,47]. If the required regulatory and public health information, including safety and efficacy data from the Phase III program, are deemed satisfactory, the WHO has indicated that a policy recommendation for the RTS,S malaria vaccine candidate is possible as early as 2015, paving the way for decisions by African nations regarding large-scale implementation of the vaccine through their national immunization programs [48]. The type of estimates generated by the MVM will be of great interest to policymakers at both the global and country levels to inform decisions around RTS,S.

### Model design

The balancing of a comprehensive approach with simplicity can lead to models that attempt to include all considerations and empirical field data, yet lose their usability and interpretability. On the other hand, a complex, vector-borne, parasitic disease, like malaria, is not well-reflected by overly simplified assumptions. Collaborators settled on a compromise to integrate the strengths of a comprehensive and computationally intensive model associating epidemiological patterns and vaccine characteristics with impact from Swiss TPH with simpler, specially designed, component modules for less computationally intensive elements such as the number of individuals that might be immunized. The MVM integrates pre-defined inputs from Swiss TPH with the other modules through a tailor-made user interface, providing a seamless means of inputting data and generating outputs.

While the MVM was designed to allow users the flexibility to enter as much of their own data as possible and choose between various input options described in Tables 1-5, the limitation is that only the simulations run by Swiss TPH can be accessed by users. It is possible for the MVM developers to upgrade the model with additional simulations, as is currently planned. The work required to design a model that is both comprehensive and easy to use should not be underestimated, however. Another limitation of the model is that it requires a large amount of storage (2.98 gigabytes), by today’s standards, on the user’s computer, the majority of which (90%) is used to hold the Swiss TPH simulation reference data.

### Uses and outputs

The MVM interface provides an accessible means to enter data and generate results, which are presented in straightforward outputs. The outputs of the MVM were designed to address questions arising from three distinct audiences: country policymakers, regional and global policymakers, and vaccine developers. The MVM can provide country policymakers with estimates of impact and costs of malaria vaccines tailored to their local transmission setting, and based on local assumptions about implementation, such as delivery strategy, coverage, and cost. One strength of the MVM is the simultaneous generation of estimates for individual and multiple countries, which regional and global policymakers may use to support the setting of standards on the use of a malaria vaccine. In addition to the graphical data in the interface, both reference data and model result reports can be easy downloaded into Excel or pdf files. The types of outputs generated by the MVM are relevant to policymakers seeking information on the role of malaria vaccines as complements to other strategies to address malaria. For malaria vaccine developers and global organizations, the MVM can help inform trade-offs between various product characteristics, delivery options, impact, and costs, as well as supply and demand considerations.

### Parameters

The MVM includes pre-entered reference parameters where possible, while allowing users the flexibility to enter a wide range of scenario-specific parameters when desired. The number of parameters and associated ranges led to more than 100,000 scenarios from Swiss TPH. It should be noted that, while all-cause childhood mortality is not an explicit component of the MVM, changes in all-cause mortality can be accounted for using the regularly updated demographic data. A number of the parameters and associated assumptions in the MVM merit specific discussion.

There is no universally agreed-upon means for converting malaria transmission data from WHO and the Malaria Atlas Project into EIR for each country, though much progress has been made recently in understanding the relationship between different measures of transmission [36]. Furthermore, there is no standard for projecting how transmission may change for each country. MVI sought the participation and validation of expert collaborators and arrived at an approach, as described in Additional file 3, which transparently translates existing data on the percentage of the population at risk into EIR-equivalents. Users are able to customize assumptions of underlying and changing transmission. Perhaps in the future there will be greater standardization on one means of measuring the prevalence and transmission of malaria.

One of the strengths brought by building upon the Swiss TPH model is that it is parameterized with extensive field data on malaria. There is always uncertainty in models, the quantification of input parameters and assumptions of their interrelations, which is not straightforward. For instance, mathematical models of malaria vaccines (Swiss TPH and Imperial College London), currently assume that efficacy decays exponentially. It is yet not established whether an exponential model reflects the decay in true vaccine protection.

Faced with multiple public health interventions and vaccines, and given the current funding environment, health economic data is becoming an increasingly important consideration in adoption decisions. The WHO uses cost-effectiveness thresholds based on gross domestic product to evaluate interventions [49], and any malaria vaccine will need to be evaluated in light of such thresholds. The price of new vaccines, rather than the cost of implementation, has been found to be a major driver of cost-effectiveness [50]. The vaccine price of $5 a dose was selected as the input for the demonstration scenario because it is consistent with the price of other novel vaccines (as there is no price yet determined for any potential malaria vaccine), but it will be critical that the cost borne by countries fit into malaria budgets that includes other type of interventions.

The purpose of the demonstration scenario presented in this paper was to illustrate the functionality of the model, rather than to forecast when individual countries might adopt a malaria vaccine. That said, the timing of implementation by countries, and time to maximum coverage, are important drivers of impact. Figure 2 illustrates the degree to which the rate of demand for doses can vary after a new vaccine becomes available. The vaccine adoption decisions of large countries, particularly those that also have a large disease burden, can greatly influence estimates of supply and demand, public health impact, and financial requirements. For example, if Nigeria had implemented the vaccine in the 8th year of its availability (rather than the 5th, as modeled), 18% fewer cases and 15% fewer deaths would have been averted overall in the demonstration scenario.

## Conclusion

The field of malaria is rapidly changing, with malaria transmission decreasing, partly due to other effective interventions, and the potential for an efficacious vaccine on the horizon. These changes are important to those developing malaria vaccines as well as those who make policy decisions on the use and financing of vaccines. This paper presents a new model on supply, demand, public health impact, and costs of malaria vaccines to inform this changing field. The model is designed to be user-friendly, is built upon the Swiss TPH model simulations, and takes into account the questions of interest to policymakers at the country, regional, and global levels, as well as vaccine developers. Results from a demonstration scenario indicate that a hypothetical vaccine with an efficacy of 85% against clinical disease could have substantial public health impact. Following validation exercises, the current MVM, future iterations, and other models like it could provide additional insight to ensure investments are well-targeted and could then inform the critical public health decisions that policymakers at national, regional, and global levels must make on the optimal means to prevent the millions of malaria cases each year.

## Competing interests

CS is employed by Applied Strategies. JN, VC, CL, NM, TS, KM, and AB have declared that no competing interests exist.

## Authors’ contributions

JN and AB drafted the manuscript. JN generated the model results. NM, TS, KM, and AB were involved in the design and/or analysis of the first version of the MVM as the foundation for the version presented here. KM’s contributions were made largely while a student at the London School of Hygiene and Tropical Medicine. VC, CL, NM, TS, CS, and AB were involved in the design and development of the current version of the MVM. All authors contributed to, reviewed, and approved the manuscript.

## Pre-publication history

The pre-publication history for this paper can be accessed here:

http://www.biomedcentral.com/1471-2334/13/295/prepub

## Supplementary Material

Additional file 1 Reference data for population, vaccine coverage, malaria transmission, and disease burden. Click here for file

Additional file 2 **Hib adoption.** Year of Hib adoption and time to maximum coverage, WHO data and adapted for demonstration scenario. Click here for file

Additional file 3 Methodology for converting WHO estimates to EIR. Click here for file

Additional file 4 **Seed count.** Reference data from Swiss TPH on vaccine impact: seed count for siumulations. Click here for file

Additional file 5 **Vaccine_Strategy 0.** Reference data from Swiss TPH on vaccine impact: Vaccine Strategy 0. Click here for file

Additional file 6 **Vaccine_Strategy 1.** Reference data from Swiss TPH on vaccine impact: Vaccine_Strategy 1. Click here for file

Additional file 7 **Vaccine_Strategy 3.** Reference data from Swiss TPH on vaccine impact: Vaccine_Strategy 3. Click here for file

Additional file 8 **Vaccine_Strategy 4.** Reference data from Swiss TPH on vaccine impact: Vaccine_Strategy 4. Click here for file

Additional file 9 **Vaccine_Strategy 5.** Reference data from Swiss TPH on vaccine impact: Vaccine_Strategy 5. Click here for file

Additional file 10 **Vaccine_Strategy 6.** Reference data from Swiss TPH on vaccine impact: Vaccine_Strategy 6. Click here for file

Additional file 11 **Vaccine_Strategy 9.** Reference data from Swiss TPH on vaccine impact: Vaccine_Strategy 9. Click here for file

Additional file 12 **Vaccine_Strategy 10.** Reference data from Swiss TPH on vaccine impact: Vaccine_Strategy 10. Click here for file

Additional file 13 **Vaccine_Strategy 13.** Reference data from Swiss TPH on vaccine impact: Vaccine_Strategy 13. Click here for file

Additional file 14 **Vaccine_Strategy 14.** Reference data from Swiss TPH on vaccine impact: Vaccine_Strategy 14. Click here for file

Additional file 15 **Vaccine_Strategy 16.** Reference data from Swiss TPH on vaccine impact: Vaccine_Strategy 16. Click here for file

Additional file 16 **Vaccine_Strategy 18.** Reference data from Swiss TPH on vaccine impact: Vaccine_Strategy 18. Click here for file

Additional file 17 **Demonstration scenario data.** Analysis of MVM outputs for demonstration scenario. Click here for file
