# Supplementary material for: Modeling the public health impact of malaria vaccines for developers and policymakers
Source: BMC Infect Dis. 2013 Jul 1;13:295. doi: 10.1186/1471-2334-13-295 (PMC3711926; doi:10.1186/1471-2334-13-295)
Supplement: Additional file 3 — Methodology for converting WHO estimates to EIR. [file 1471-2334-13-295-S3.doc]

# Additional file 3 – Methodology for converting WHO estimates to EIR

In order to provide public health impact estimates, the Swiss Tropical and Public Health Institute (Swiss TPH) model requires malaria transmission data in entomologic inoculation rate (EIR) format. As such, the PATH Malaria Vaccine Initiative (MVI) created the following five EIR buckets (or categories) for grouping data:

- A: intended to approximate an EIR of 0
- B: intended to approximate an EIR of 0.1
- C: intended to approximate an EIR of 1.0
- D: intended to approximate an EIR of 10.0
- E: intended to approximate an EIR of 100

The World Health Organization (WHO) did not provide their transmission data in EIR format. The need then, was to create a distribution of each country’s population across these five EIR buckets, such as depicted here. These distributions are updated on annual basis as the most current WHO World Malaria Report becomes available.


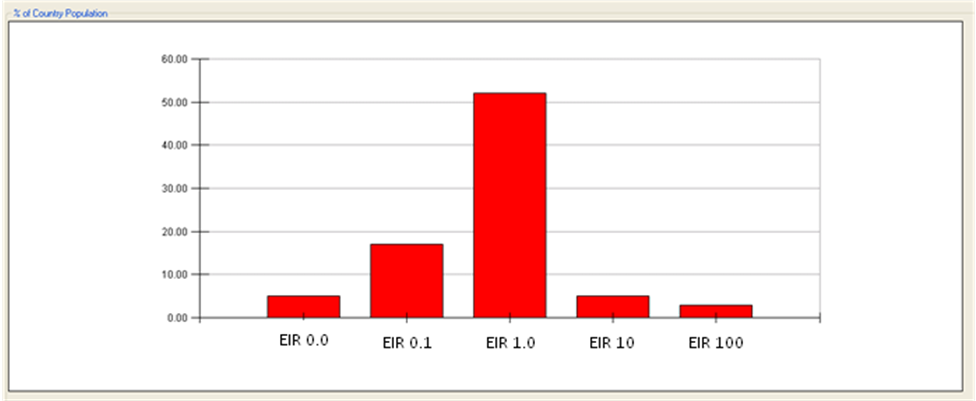


The goal was to find the best representation of transmission risk—in EIR format—for each country.

# WHO-based transmission scenario

For the WHO-based transmission scenario, MVI elected to create distributions such that simulated (i.e. modeled) disease burden approximated WHO disease burden, as shown below.


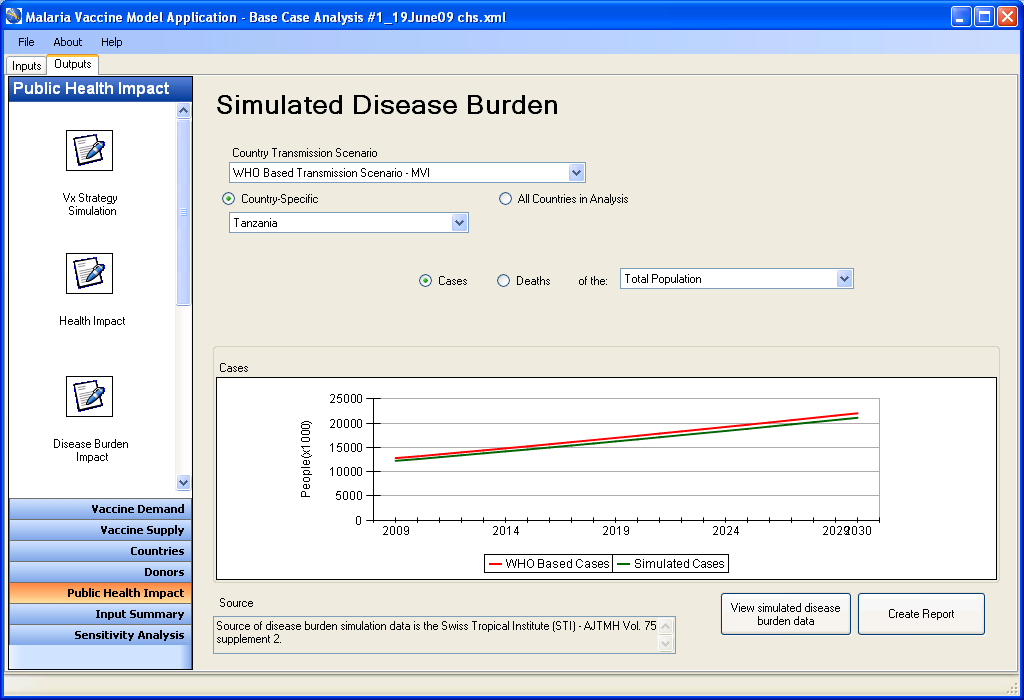


In the WHO *World Malaria Report 2008* (WMR), for example, WHO expressed transmission risk as the percentage of population in each country at:

- high risk of transmission
- low risk of transmission

For some countries, the total percentage reported did not amount to 100%. In these cases, it was assumed that the missing percentage is not endemic for malaria. WHO data also included the %*P. falciparum* (*Pf*) in each country (as opposed to *P. vivax (Pv)*).

**E.g. Tanzania**

- **High transmission: 75%**
- **Low transmission: 25%**

**(assume 0% is non-endemic for malaria)**

- **%*Pf*: 100%**

WHO-based transmission scenario methodology

High and low transmission percentages for each country were multiplied by the *Pf* percentage to arrive at the high and low transmission that is specific for *Pf* for each country. This assumes that *Pf* and *Pv* are similarly distributed in each country—an assumption made for lack of information to the contrary.

**E.g. Tanzania**

- **High transmission: 75% x 100% = 75%**
- **Low transmission: 25% x 100% = 25%**

Countries were then ranked by the total cases per 100,000 people (as provided in the WMR) and divided into the following 13 categories:

- 0 – 400 cases/100,000
- 400 – 900 cases/100,000
- 900 – 1500 cases/100,000
- 1500 – 2000 cases/100,000
- 2000 – 5000 cases/100,000
- 5000 – 10000 cases/100,000
- 10000 – 16000 cases/100,000
- 16000 – 25000 cases/100,000
- 25000 – 30000 cases/100,000
- 30000 – 34000 cases/100,000
- 34000 – 38000 cases/100,000
- 38000 – 41000 cases/100,000
- 41000+ cases/100,000

Within each of the 13 categories, each country’s population was distributed across each of the five EIR buckets for the two levels of transmission risk (high and low). As a reminder, the goal was to determine the distribution that best approximates WHO disease burden. Through trial and error, distributions were set for each of the 13 categories.

**E.g. 25,000-30,000**

All countries falling into this category were ascribed the same distribution for low and high transmission.

|  | High transmission | Low transmission |
| --- | --- | --- |
| EIR 0.0 | 47.5% | 52.0% |
| EIR 0.1 | 25.0% | 25.0% |
| EIR 1.0 | 20.0% | 20.0% |
| EIR 10 | 6.0% | 3.0% |
| EIR 100 | 1.5% | 0.0% |

**Note**: The EIR 0.0 category is not actually assigned, rather it receives the unassigned distribution. In other words, it is presumed to be the difference between 100% and the sum of the four other buckets (i.e. EIR 0.0 = (100 – (EIR 0.1 + EIR 1.0 + EIR 10.0 + EIR 100.0)).

For example, according to WHO, Tanzania had a disease burden of 29,245 cases per 100,000 population. As a result, Tanzania falls into the 25,000 – 30,000 cases/100,000 category, as defined above.

For each EIR bucket, the distributions were weighted by the high and low transmission risk percentages for that country, and then summed across the high and low transmission settings.

**E.g. Tanzania**

|  | High transmission | Low transmission | Sum |
| --- | --- | --- | --- |
| EIR 0.0 | 47.5% *** 75%** | 52.0% *** 25%** | **35.6 + 13.0 = 48.6%** |
| EIR 0.1 | 25.0% *** 75%** | 25.0% *** 25%** | **18.8 + 6.3 = 25%** |
| EIR 1.0 | 20.0% *** 75%** | 20.0% *** 25%** | **15.0 + 5.0 = 20.0** |
| EIR 10 | 6.0% *** 75%** | 3.0% *** 25%** | **4.5 + .8 = 5.3** |
| EIR 100 | 1.5% *** 75%** | 0.0% *** 25%** | **1.1 + 0.0 = 1.1** |

| **% of Population in EIR = 0** | **% of Population in EIR = 0.1** | **% of Population in EIR = 1.0** | **% of Population in EIR = 10** | **% of Population in EIR = 100** |
| --- | --- | --- | --- | --- |
| 49% | 25% | 20% | 5.3% | 1.1% |

The WHO-based transmission scenario holds the transmission risk constant over time. However, users are free to create scenarios in which transmission risk varies over time or in which views about transmission risk varies across countries. Applying the country’s population to the distribution in the transmission scenario provides the modeled disease burden over time. This modeled disease burden may then be compared to the expectation of the WHO disease burden, as shown here for Tanzania, in a screen shot from the Malaria Vaccine Model.


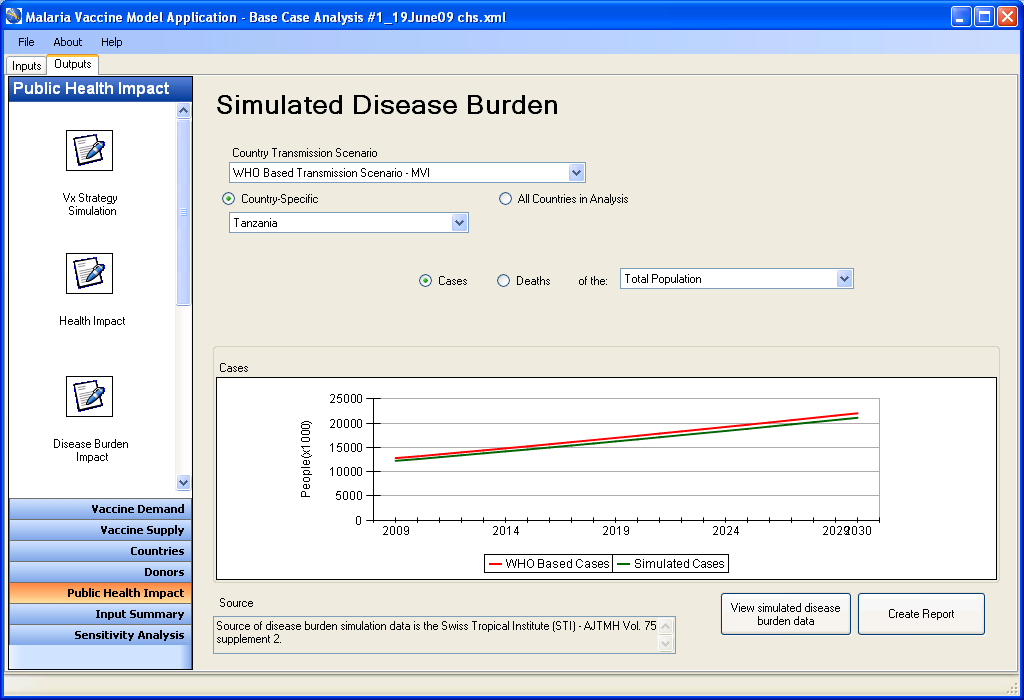


It is important to note that this WHO-based transmission scenario is but one representation of how low and high transmission risk may be translated to EIR. Users are free—and encouraged—to create their own transmission scenarios.

**Note**: Transmission scenarios require significant computer memory. The user is advised to keep this in mind when creating multiple scenarios.
